# Improving the predictability of rotational correction with clear aligners: Evidence gaps, biomechanical principles, and clinical strategies

**DOI:** 10.1016/j.jobcr.2026.101567

**Published:** 2026-10-30

**Authors:** Preeti Kulkarni, Puneet Batra, Nikhillesh Vaiid

**Affiliations:** a Department of Orthodontics & Dentofacial Orthopaedics, Manav Rachna Dental College, Manav Rachna International Institute of Research and Studies (MRIIRS), Faridabad, India; b School of Dental Sciences, School of Allied Health Sciences, Department of Orthodontics and Dentofacial Orthopaedics, Manav Rachna International Institute of Research and Studies (MRIIRS), Faridabad, India; c Department of Orthodontics, University of Illinois Chicago College of Dentistry, Chicago, IL, 60607, USA; d Saveetha Institute of Medical and Technical Sciences, Chennai, India; e Paediatric Dentistry and Orthodontics, Faculty of Dentistry, University of Hong Kong, Hong Kong SAR, China

**Keywords:** Clear aligners, Tooth rotation, Orthodontic biomechanics, Attachments, Treatment predictability, Treatment outcome

## Abstract

Clear aligner therapy (CAT) has evolved from an aesthetic modality into a widely used orthodontic treatment system capable of addressing increasingly complex malocclusions. Improvements in thermoplastic materials, digital planning, staging protocols, and attachment design have broadened the scope of treatment; however, rotational correction remains one of the least predictable tooth movements. The expression of derotation is influenced by tooth morphology, rotational direction, magnitude of correction, available space, staging velocity, attachment geometry, wear compliance, and unintended three-dimensional side effects. Yet much of the available evidence continues to evaluate rotation as a single endpoint, thereby limiting the clinical utility of reported predictability estimates.

This narrative review synthesises clinical, systematic-review, and biomechanical evidence on rotational correction with clear aligners, emphasising movement-specific determinants, evidence limitations, and practical management strategies. The available literature suggests that derotation may be more predictable when treatment planning accounts for tooth-specific anatomy, movement magnitude and direction, space availability, staging strategy, attachment design, and treatment compliance. For severe or resistant rotations, adjunctive or hybrid mechanics may be considered when adequate expression cannot be achieved with aligner-only mechanics. Overall, a movement-specific and biomechanics-informed approach may help improve the clinical management of rotational correction, although further prospective evidence is needed to establish the relative contribution of individual treatment variables.

## List of abbreviations:

**CAT**: Clear aligner therapy
**IPR**: Interproximal reduction
**TAD**: Temporary anchorage device
**FEA**: Finite element analysis

## 1 Introduction

CAT has expanded from the treatment of mild malocclusions to increasingly complex orthodontic problems. Nevertheless, the accuracy of clinically achieved tooth movement remains variable, particularly for biomechanically demanding movements. Rotational correction is consistently reported among the less predictable movements, with discrepancies between digitally programmed and clinically achieved outcomes. Advances in intraoral scanning, virtual treatment planning, material science, attachment design, and staging algorithms have expanded the biologic and mechanical possibilities of aligner therapy.^1^, ^2^, ^3^, ^4^ Contemporary scoping reviews of the clear aligner literature confirm that the volume of published evidence has grown accordingly, even though methodological quality and level of evidence remain uneven across studies, and treatment outcome remains the most frequently reported but least rigorously standardised domain.^1^ Commentary within the speciality has repeatedly cautioned against outpacing the evidence base with new aligner technologies, and has highlighted a persistent gap between digitally anticipated outcomes and what is actually achieved clinically; a gap that is often widest for the more biomechanically demanding movements.^2^, ^3^

Even so, not all tooth movements have benefited equally from these developments. Rotational correction remains one of the most technically demanding and least predictable objectives in aligner-based treatment.^5^, ^6^, ^7^ This limitation is not merely a matter of appliance design. Tooth morphology, root form, crown curvature, tooth type, rotation direction and magnitude, interproximal space, staging strategy, compliance, and the three-dimensional force system generated by the aligner–attachment interface all influence rotational control. Many reports treat rotation as a simple angular endpoint; this simplification risks obscuring clinically important variation in predictability across tooth types and movement directions. A more useful interpretation requires attention to biomechanical context rather than reliance on digital setup alone. Such interpretation also requires critical appraisal of heterogeneous evidence and its translation into clinically reasoned decision-making rather than the uncritical application of reported predictability values.^7^, ^8^ This narrative review critically examines the determinants and limitations of rotational correction with clear aligners and translates the available evidence into a practical clinical decision framework.

## 2 Review approach

This narrative review was developed through a literature-based approach involving searches of MEDLINE and Scopus, critical reading of relevant publications, and reference tracking. The literature search focused on clear aligner therapy, rotational tooth movement, derotation, attachment design, staging protocols, interproximal reduction, and orthodontic biomechanics. The search was exploratory, using combinations of topic-related terms rather than a predefined, standardised Boolean strategy. Exact historical database queries, retrieval counts, and the final search date were not retained. No predefined publication-year or language filters were applied during database searching. During literature selection, emphasis was placed on recent, relevant evidence; the publications ultimately included in this review were published from 2016 onwards.

Publications were considered for inclusion based on their relevance to the predictability, biomechanical determinants, and clinical management of rotational correction with clear aligners. Relevant clinical investigations, systematic reviews, and laboratory and finite element analysis (FEA) studies were considered to provide complementary perspectives. Publications without a meaningful relationship to rotational correction or its underlying biomechanics were excluded. Reference lists of relevant publications were examined to identify additional studies.

Literature identification, selection, and critical evaluation were undertaken primarily by the lead author, with additional relevant publications suggested by the co-authors during manuscript development. Suggested references were reviewed by the lead author for their relevance, methodological characteristics, and contribution to the objectives of the review before inclusion. Study selection was guided by thematic relevance and contribution to the clinical or biomechanical understanding of rotational correction rather than by a formal systematic screening protocol. As independent multi-reviewer screening was not undertaken, no formal inter-reviewer agreement assessment or predefined disagreement-resolution procedure was applied.

The selected literature was evaluated through critical reading and narrative synthesis, consistent with the interpretive approach of narrative reviews.^9^ The methodological workflow involved identifying relevant publications, assessing their relevance to the review objectives, examining study designs and reported findings, and organising the evidence into recurring themes. These themes included tooth morphology, movement magnitude and direction, space availability, attachment design, staging strategies, and adjunctive mechanics. Particular attention was given to differences in study design, aligner systems, movement characteristics, outcome measurements, and methodological limitations. Methodological limitations and potential sources of bias were considered qualitatively; no formal study-level risk-of-bias assessment using a standardised instrument was performed.

Clinical investigations were considered primarily for evidence regarding achieved tooth movement, treatment predictability, and clinical outcomes. Laboratory and FEA studies were examined for their contributions to understanding force systems, initial displacement patterns, and biomechanical behaviour. Findings from laboratory and FEA investigations were interpreted as mechanistic evidence rather than direct confirmation of clinical effectiveness.

As this was a narrative review, no formal systematic-review protocol, independent multi-reviewer screening, or quantitative meta-analysis was undertaken. The proposed clinical strategies represent evidence-informed interpretations of the available literature rather than independently validated treatment protocols.

The review was restricted to published, peer-reviewed journal articles and reviews. No separate systematic search of grey literature, including unpublished dissertations, conference abstracts, or preprints, was undertaken. This may have limited the identification of unpublished or emerging evidence. The literature identification, selection, and appraisal approach, together with its limitations, is summarised in Supplementary Table S1.

## 3 Evidence gaps in rotational correction

### 3.1 Direction-specific rotation patterns

The available evidence indicates that rotational predictability is not uniform across movement directions or tooth groups. Rather, derotation appears to depend on the interaction between crown morphology and the direction of force application. Retrospective cohort data that model direction-dependent expression show that the same nominal rotation can track differently depending on whether the tooth is rotated in opposing directions around its long axis, defined relative to the tooth and viewed from a specified reference plane. This is not captured when studies report a single mean predictability value per tooth type. Direction-specific analyses may therefore provide a more clinically informative assessment than reporting a single mean predictability value for each tooth type.^6^, ^10^, ^11^

### 3.2 Tooth morphology and tooth type

Canines and premolars remain disproportionately resistant to complete derotation. This may be attributed to a combination of morphological and biomechanical factors. The relatively rounded crown contours of premolars and the convex labial surfaces of canines can limit effective aligner engagement, reducing the efficiency of rotational force transmission. Inadequate engagement may result in aligner deformation or slippage rather than complete expression of the programmed movement. Furthermore, the location and direction of force application influence the generation of an effective rotational moment around the tooth's long axis, while variations in root morphology and resistance from adjacent proximal contacts may further affect rotational response. Tooth type alone, however, is an incomplete predictor; individual crown and root morphology, resistance form, and biomechanical conditions are equally relevant. Available evidence indicates that rotational predictability varies considerably even within the same tooth type.^5^, ^6^, ^12^

### 3.3 Space availability and the role of interproximal reduction (IPR)

Interproximal space is a fundamental determinant of rotational expression. In crowded segments, aligners may fail to engage the tooth adequately, limiting the transmission of the intended force system. IPR may provide clearance for derotation when interproximal space is limiting, but evidence that IPR independently improves rotational accuracy remains limited. Prospective follow-up data suggest that the benefit of IPR is most apparent when space deficiency, rather than staging or attachment design, is the primary limiting factor.^13^, ^14^, ^15^

### 3.4 Staging velocity and per-stage limits

The magnitude of movement programmed into each aligner stage appears to influence tracking accuracy. Larger programmed increments have been associated with reduced expression, although the optimal per-aligner rotational increment remains insufficiently established clinically. A recent retrospective clinical investigation of maxillary canine derotation further reported an association between greater programmed rotational velocity per aligner and increased rotational under-expression, providing additional tooth-specific clinical evidence for the relevance of staging magnitude.^16^ Conservative staging has been associated with more favourable tracking in available studies; however, staging velocity is rarely evaluated as an independently manipulated variable in clinical outcome research. Treatment-design comparisons and reviews of macro- and micro-staging concepts therefore support consideration of smaller rotational increments, particularly for more challenging movements, while the magnitude of their independent clinical benefit remains uncertain.^13^, ^17^, ^18^, ^19^.

### 3.5 Crown-root and adjacent-tooth effects

Rotational correction is rarely isolated. The force systems used to derotate a tooth may also induce tipping, translational displacement, or unwanted effects in adjacent teeth. Biomechanical reviews of aligner force systems confirm that clinically generated aligner force systems rarely behave as ideal pure couples; concomitant forces and moments may therefore produce tipping, translation, intrusion, extrusion, or effects on adjacent teeth (Fig. 1). These collateral movements are clinically relevant, particularly when treatment success is judged by crown alignment alone rather than by full three-dimensional expression.^20^, ^21^

**Fig. 1:**
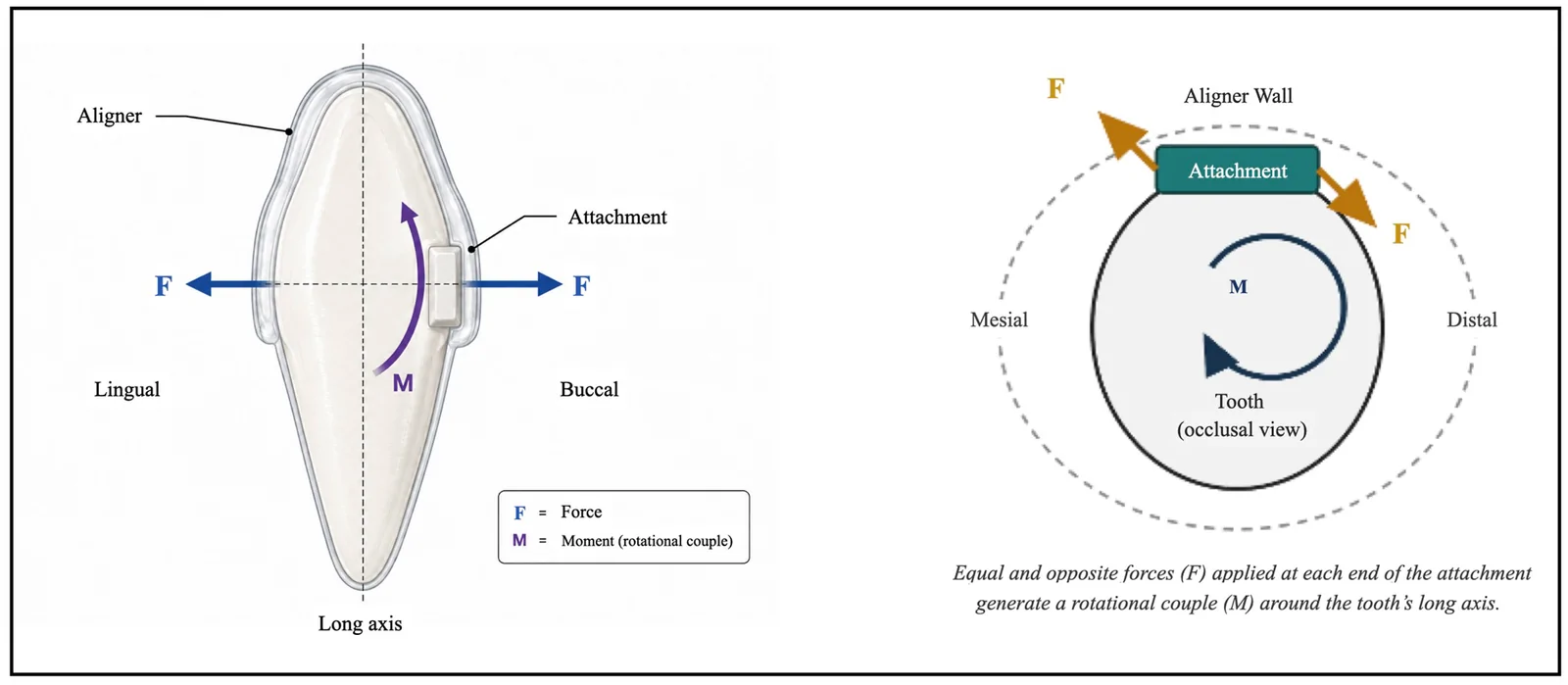
Biomechanical basis of a rotational force couple. Equal and opposite forces applied at each end of the attachment generate a rotational moment (M) around the tooth's long axis.

### 3.6 Clinical contribution of attachments

Attachment design remains one of the most debated variables in aligner biomechanics. Although optimised attachments may improve force transmission in some contexts, the evidence does not support a universal advantage; retrospective comparisons of attachment efficacy across movement types have found benefits that are inconsistent and, in several instances, not statistically significant once movement type is controlled for. Their value appears to be movement-specific, anatomy-dependent, and highly contingent on the broader treatment design rather than an independent predictor of success.^22^, ^23^, ^24^

An important distinction should be maintained between clinical evidence of attachment effectiveness and biomechanical findings derived from laboratory or finite element investigations. While FEA studies can demonstrate differences in initial tooth displacement, force distribution, and periodontal stress under simulated conditions, these outcomes do not directly establish the clinical accuracy or long-term effectiveness of rotational correction. Clinical studies remain necessary to determine whether biomechanical advantages observed experimentally translate into improved treatment outcomes.

### 3.7 Wear protocol, compliance and treatment delivery

Wear time, aligner replacement interval, and attachment protocol all influence how much planned rotation is expressed clinically. Cohort data evaluating attachment and wear protocols for canine derotation suggest that treatment-delivery variables may influence the clinical expression of programmed rotation.^25^ Compliance should therefore be considered alongside the biomechanical prescription when interpreting treatment fidelity.

### 3.8 Stability, refinement burden, and outcome interpretation

Many studies focus on initial tracking rather than on refinement burden, treatment efficiency, or long-term stability, limiting the clinical interpretation of reported predictability outcomes.^7^ As a result, apparent treatment success may not adequately reflect the clinical cost of achieving the final outcome, including additional aligner series, prolonged treatment duration, and increased patient burden.^26^ Systematic-review evidence has identified multiple clinical, biomechanical, and digital factors associated with the need for refinement after the first series of clear aligners, while tooth-level analyses have further demonstrated that refinement requirements vary according to tooth- and movement-specific characteristics.^27^, ^28^ Long-term stability represents an additional dimension of treatment success that is not captured by first-series tracking or immediate post-treatment accuracy alone.^28^ Finally, heterogeneity in study design, outcome definitions, measurement methods, and analytical approaches reduces comparability across studies and limits the confidence with which reported predictability estimates can be interpreted or pooled.^7^, ^29^ A recent systematic review focusing specifically on rotational tooth movements with clear aligners similarly highlighted substantial variability in reported rotational accuracy and limitations in the certainty of the available evidence, further emphasising the need for standardised, tooth-specific and movement-specific outcome assessment.^6^

## 4 Clinical principles for more predictable rotational control

The following clinical principles are derived from an interpretation of the available clinical and biomechanical literature. They should be regarded as evidence-informed considerations rather than universally validated treatment protocols. The strength of supporting evidence varies across individual strategies, and their application should be guided by tooth morphology, movement requirements, clinical circumstances, and professional judgement.

### 4.1 Anticipate under-expression, particularly in canines and premolars

Rotational movements may demonstrate incomplete clinical expression more frequently than relatively simpler movements such as alignment or mild tipping.^6^ This limitation is particularly relevant in teeth with less favourable rotational morphology, including canines and premolars, where tooth shape and movement-related factors can reduce predictability.^6^, ^10^ Furthermore, rotational expression may vary according to the direction of the programmed movement**,** reinforcing that predictability cannot be considered uniform even within the same tooth type.^12^ Clinicians should therefore anticipate possible under-expression and incorporate this into treatment planning, patient expectations, and refinement strategies.

### 4.2 Use smaller staging increments

Conservative rotational staging may improve aligner tracking, particularly for canines, premolars, and teeth requiring substantial derotation. An in vitro investigation of premolar rotation reported that programmed rotational increments exceeding 1° generated high moments, providing a biomechanical rationale for considering smaller increments in challenging rotations. However, this experimental observation does not establish a clinically validated staging threshold. Reducing the programmed movement per stage may help moderate the forces and moments generated at the aligner–tooth interface, although its independent effect on clinical rotational accuracy remains uncertain. The appropriate staging increment should therefore be individualised according to tooth morphology, rotation magnitude, available space, attachment configuration, and clinical response. Further prospective clinical investigations are needed to determine the optimal rotational increment per aligner.^13^, ^16^, ^17^, ^18^, ^19^, ^30^.

### 4.3 Consider deliberate overcorrection for severe rotations

Where rotation is pronounced, planned overcorrection may be considered to compensate for anticipated under-expression.^12^ The need for such compensation should be assessed in relation to tooth type, morphology, magnitude of planned derotation, and available space, as these factors can influence the predictability of rotational movement.^12^, ^14^ Overcorrection should therefore be applied judiciously and within a sound biomechanical plan rather than used routinely as a substitute for adequate space, staging, or force-system control.

### 4.4 Favour double attachments in difficult premolar rotations when indicated

For selected premolar rotations, double attachments may provide greater rotational control than a single-attachment configuration. A retrospective comparison of single-versus double-attachment protocols demonstrated more complete clinical expression of programmed premolar rotation with the double-attachment approach.^31^ FEA further indicates that attachment position and the distribution of force application influence the rotational moment generated at the aligner–tooth interface.^32^ Similarly, biomechanical analysis of canine derotation has shown that increasing the number and location of active force-application surfaces can enhance rotational displacement, although this may also increase periodontal and bone stress.^32^ Collectively, these findings provide a biomechanical rationale for multi-surface engagement in selected difficult rotations but do not establish a universally superior attachment configuration. Attachment selection should therefore remain case-specific rather than routinely favouring double attachments.

### 4.5 Do not assume that optimised attachments are always superior

The available evidence does not support a blanket assumption that optimised attachments consistently outperform conventional attachment designs across all orthodontic movements.^22^, ^23^ Systematic-review evidence suggests that any advantage of optimised attachments is movement-specific and dependent on the biomechanical context, rather than universally applicable.^22^ For rotational movements in particular, clinical data have not demonstrated a consistent superiority of optimised over conventional attachments, indicating that attachment optimisation alone does not guarantee improved rotational expression.^24^ Attachment selection should therefore be based on the specific tooth, planned movement, and required force system rather than on the assumption that an optimised design is inherently more effective.

### 4.6 Choose attachment geometry intentionally

Attachment geometry influences the force system delivered to the tooth and, consequently, the quality of rotational control. FEA of canine derotation demonstrates that different attachment configurations can alter rotational displacement and associated periodontal and bone stress distribution.^33^ The broader influence of attachment geometry on aligner biomechanics is supported by finite-element studies of other tooth movements. Changes in attachment design during labio-lingual tooth movement produced different biomechanical responses,^33^ while attachment configuration during maxillary anterior intrusion influenced force transmission, tooth displacement, and anchorage control.^34^ Studies evaluating attachment size and shape during rotation, tipping, and torque similarly demonstrate geometry-dependent biomechanical effects rather than supporting a single universally optimal design.^35^ Clinical evidence comparing different attachment configurations during rotational movement further indicates that attachment design can influence the accuracy of rotational expression.^36^ An in vitro comparison further demonstrated that vertical rectangular attachments generated greater derotational moments during premolar rotation, although these were accompanied by increased unwanted forces and moments, highlighting the importance of balancing rotational effectiveness against potential biomechanical side effects.^30^ Attachment shape, orientation, and position should therefore be selected according to the intended force system, tooth morphology, and clinical situation rather than by routine preference (Fig. 2).

**Fig. 2:**
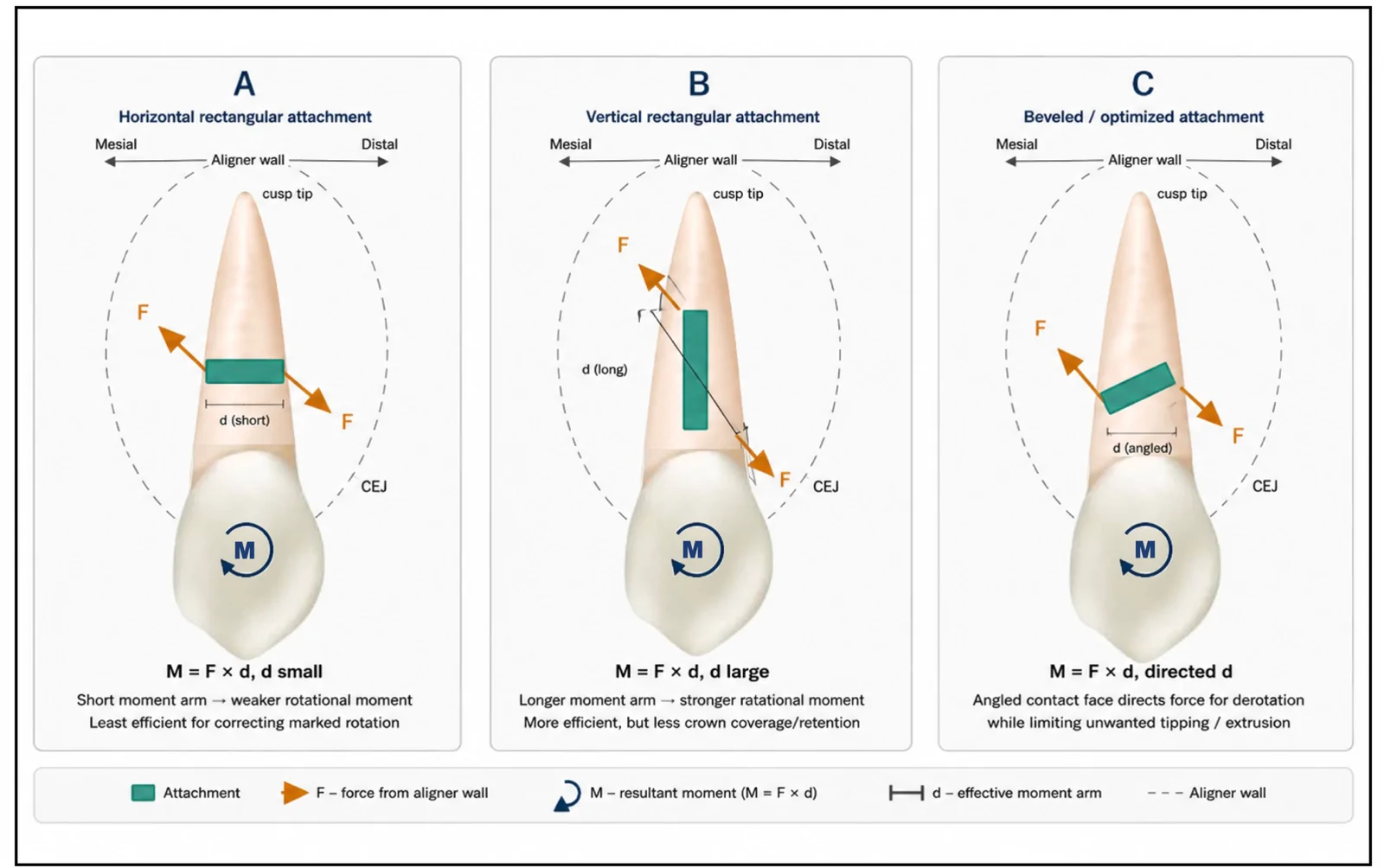
Attachment geometry changes the effective moment arm (d) and, in turn, the resultant moment (M = F x d) delivered about the canine's long axis. Buccal view, maxillary canine; attachment positioned on the middle third of the clinical crown. A: horizontal rectangular attachment (short d); B: vertical rectangular attachment (long d); C: beveled/optimised attachment (directed d).

### 4.7 Create space before attempting derotation of crowded teeth

Crowding and inadequate interproximal space can compromise the expression of rotational movements by restricting the space available for the tooth to derotate.^14^ Treatment design and the sequencing of associated tooth movements can further influence the predictability of aligner-mediated correction.^13^ In crowded situations, adequate space should therefore be created before, or appropriately staged with, active derotation rather than attempting multiple competing movements simultaneously.

### 4.8 Use IPR selectively

IPR may facilitate rotational correction when inadequate interproximal space restricts the movement required for derotation.^13^ However, available clinical evidence does not support the routine use of IPR as a means of improving rotational predictability in all cases.^15^ IPR should therefore be prescribed selectively, when space deficiency is identified as a biomechanical limitation to the planned rotation, rather than incorporated routinely into the derotation protocol.

### 4.9 Monitor for tipping and adjacent-tooth effects

Rotational correction should be evaluated as a three-dimensional tooth movement rather than solely by changes in crown position. Clear-aligner force systems may generate accompanying moments and secondary tooth movements in addition to the intended movement, making unwanted tipping and other off-target effects clinically relevant.^20^, ^21^ Assessment based only on apparent crown derotation may therefore overlook undesirable changes in tooth inclination or effects on adjacent teeth.^20^ Clinicians should monitor rotational correction in all three dimensions and modify staging or mechanics when unintended movements become evident.

### 4.10 Escalate to hybrid mechanics when aligner-only treatment is insufficient

Severe or resistant rotations, particularly in teeth with rounded morphology, may warrant early consideration of adjunctive or hybrid mechanics when predictable correction with aligners alone may be difficult to achieve.^6^ Clinical evidence specifically evaluating severe rotations of rounded teeth has demonstrated improved rotational accuracy with a hybrid approach compared with conventional aligner-based mechanics.^37^ More broadly, reports of temporary anchorage device (TAD) assisted clear-aligner mechanics and custom tunnel-attachment systems demonstrate how adjunctive strategies can modify the force system when complex tooth movements cannot be predictably achieved with the aligner alone.^38^, ^39^ When repeated loss of tracking or rotational under-expression indicates that the existing force system is inadequate, modifying the mechanics may therefore be preferable to repeatedly prescribing refinement with essentially the same biomechanical strategy.

### 4.11 Integration of clinical and biomechanical evidence

The available evidence suggests that rotational predictability cannot be attributed to any single treatment variable. Clinical studies provide information on achieved movement and treatment outcomes, whereas laboratory and finite element investigations help explain the mechanical behaviour of different attachment configurations and force systems. However, improvements in simulated rotational displacement or force transmission should not be interpreted as direct evidence of superior clinical effectiveness. Similarly, the potential benefits of conservative staging, overcorrection, and adjunctive mechanics must be considered in the context of tooth morphology, available space, treatment compliance, and the limitations of existing clinical studies. These findings support an individualised approach to rotational correction rather than reliance on a universally applicable attachment design or staging protocol (see Fig. 2).

## 5 Proposed chairside decision sequence

The principles outlined in Table 1 and Fig. 3 can be translated into a structured chairside decision sequence for rotational correction (Fig. 4). The sequence begins by defining the planned movement and ensuring adequate space, followed by appropriate staging and attachment selection. Treatment response is then monitored clinically, with modification or escalation of the force system if rotational expression becomes inadequate.

i. **Define the movement.** Consider the tooth type, crown morphology, direction, and magnitude of the planned rotation.
ii. **Ensure adequate space.** Assess whether sufficient mesiodistal space is available for derotation. If space is inadequate, create the required clearance before or appropriately staged with active rotation.
iii. **Plan staging and attachment design.** Use conservative staging and select an attachment configuration that supports the intended force system.
iv. **Consider overcorrection selectively.** Determine whether planned overcorrection is appropriate based on tooth morphology, rotational magnitude, and anticipated under-expression.
v. **Monitor three-dimensional expression.** At review visits, assess overall tracking and tooth position rather than evaluating crown derotation alone. If rotational expression is satisfactory, continue treatment as planned.
vi. **Identify the limiting factor when expression deteriorates.** Reassess space availability, aligner grip and engagement, staging, compliance, attachment/force-system design, and whether other programmed movements are competing with the intended rotation.
vii. **Revise the mechanics when failure persists.** If the same rotational movement repeatedly under-expresses despite correction of identifiable limiting factors, modify the force system rather than simply repeating the same prescription. Consider hybrid or adjunctive mechanics when indicated

**Table 1:** Current evidence gaps in rotational correction with clear aligner therapy and their clinical implications.

Evidence gap	What the literature currently shows	Potential clinical implication	References
Rotation direction	Rotational predictability may vary according to the direction of planned movement, even within the same tooth type.	Consider rotation direction during treatment planning rather than assuming equivalent predictability in both directions.	^11^
Tooth morphology	Rotational predictability varies among tooth types, with rounded teeth, particularly canines and premolars, generally demonstrating greater difficulty in achieving programmed rotation.	Anticipate greater risk of under-expression in morphologically challenging teeth and plan additional biomechanical control where indicated.	5, 6, 10, 14
Magnitude of rotation/overcorrection	Larger programmed rotations tend to demonstrate reduced predictability, although the relationship between planned magnitude and achieved movement is not uniform. Planned overcorrection has been proposed to compensate for anticipated under-expression in selected cases.	Consider conservative staging and selective overcorrection for larger rotations rather than assuming complete expression of the programmed movement.	12, 14
Space availability/IPR	Inadequate interproximal space and crowding may restrict rotational expression. IPR may facilitate derotation when space is limiting, but evidence does not support its indiscriminate use to improve rotational accuracy.	Ensure adequate space before or during appropriately staged derotation; prescribe IPR selectively when space deficiency represents a biomechanical limitation.	13, 14, 15
Attachment design	Attachment geometry, position and configuration can modify aligner–tooth engagement and the resulting force system; however, evidence does not demonstrate universal superiority of any single attachment design.	Select attachment design according to tooth morphology, planned movement and the required force system rather than routinely favouring one configuration.	22, 23, 24, 25, 31, 32, 33, 36, 40
Staging and treatment design	The magnitude, sequence and combination of programmed movements influence rotational expression. Staging appears particularly relevant for difficult rotations.	Deliberately sequence movements and avoid unnecessarily complex simultaneous movements when rotational control is critical.	13, 18, 19, 20
Refinement, efficiency and stability	First-series tracking does not fully represent overall treatment success. Refinement requirements, treatment duration and longer-term stability remain incompletely captured by conventional predictability measures.	Evaluate success beyond initial movement accuracy by considering refinement burden, overall treatment efficiency and stability.	7, 26, 27, 28, 29
Severe or resistant rotations	Aligner-only mechanics may be insufficient for selected severe or resistant rotations. Hybrid and adjunctive approaches provide alternative force systems when conventional aligner mechanics fail to achieve adequate expression.	Consider timely escalation when repeated under-expression or loss of tracking indicates that the existing aligner force system is inadequate.	6, 36, 37, 38, 39

**Fig. 3:**
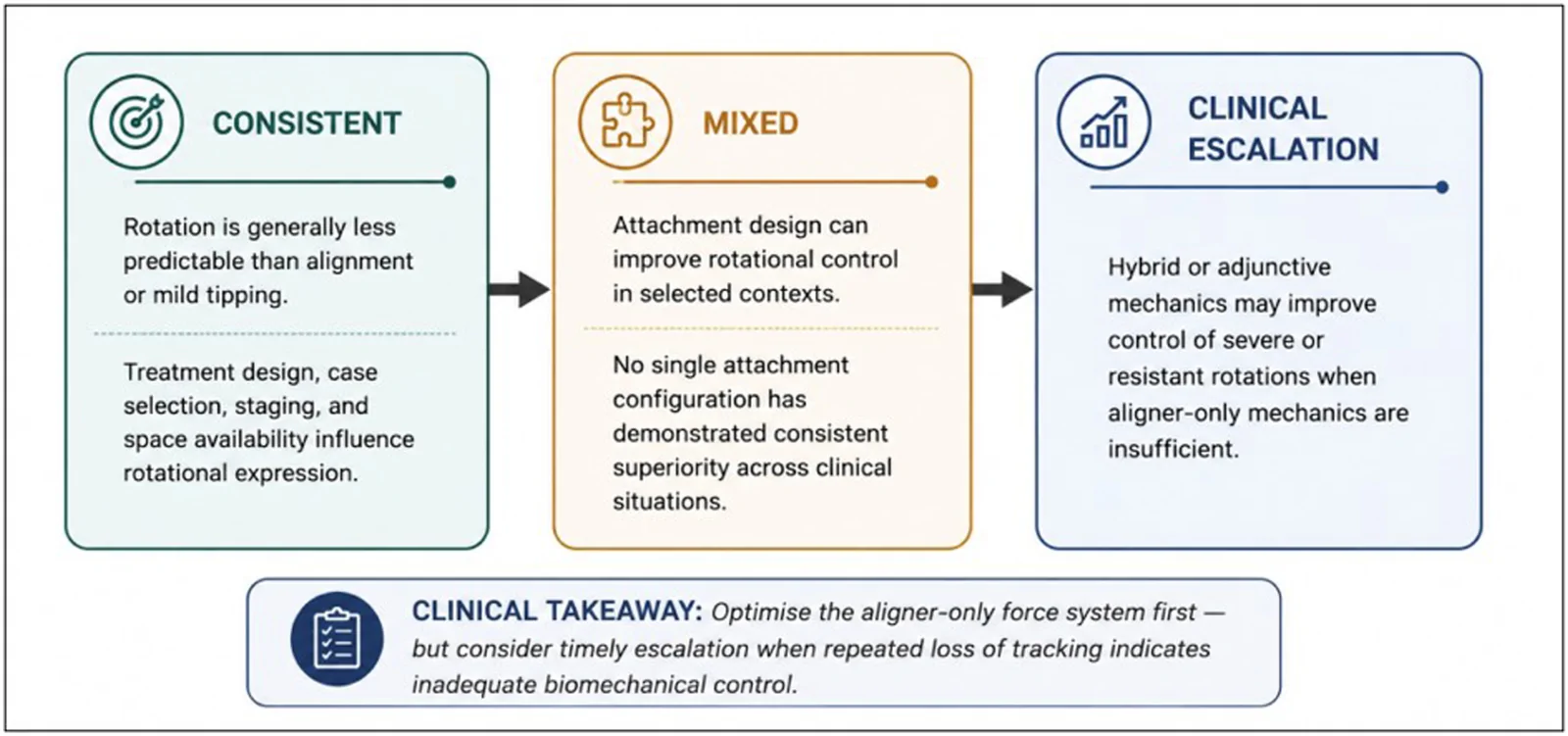
Summary of current evidence on rotational correction with CAT. Rotational movements are generally less predictable, and the effectiveness of attachment design varies across clinical situations. No single attachment design is consistently superior. For severe or resistant rotations, hybrid or adjunctive mechanics may be considered when aligner-only treatment is insufficient.

**Fig. 4:**
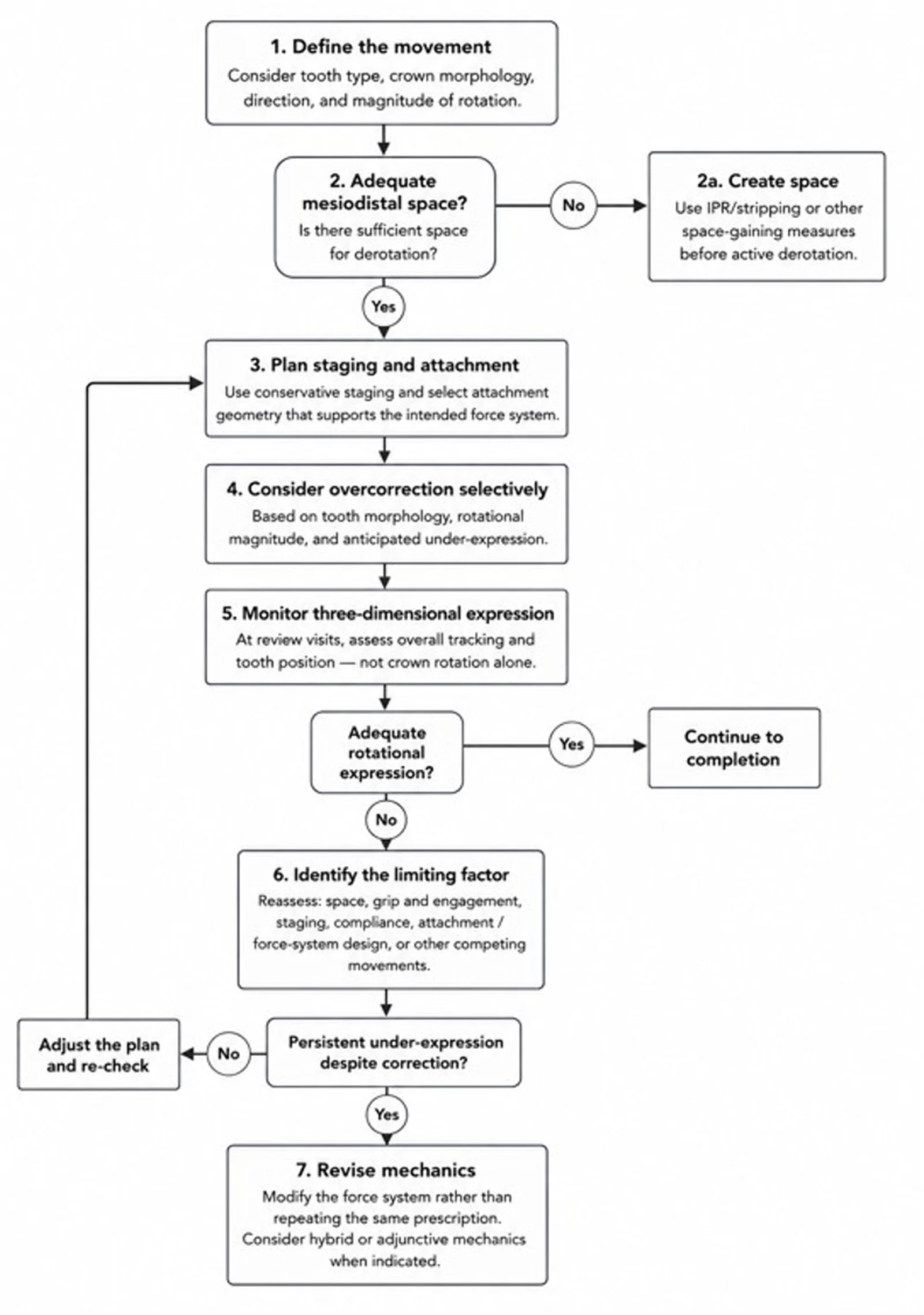
A practical chairside decision sequence for rotational correction with CAT.

## 6 Conclusion

Rotational correction remains a significant challenge in CAT, particularly for teeth with unfavourable crown morphology and for larger or mechanically demanding rotations. Current evidence indicates that predictable derotation depends on the interaction of multiple factors, including tooth morphology, movement direction and magnitude, space availability, staging, attachment configuration, and treatment compliance, rather than any single appliance modification. Clinically, this highlights the importance of individualised treatment planning, adequate space management, biomechanically appropriate attachment selection, and careful monitoring of three-dimensional tooth movement. When rotational under-expression persists, reassessment and modification of the force system, including consideration of adjunctive or hybrid mechanics, may be more appropriate than repeatedly prescribing the same movement. Nevertheless, the available evidence remains limited by methodological heterogeneity and insufficient prospective clinical validation. Future investigations should employ standardised, tooth-specific and direction-specific outcome measures and evaluate treatment efficiency, refinement requirements, and long-term stability. The proposed clinical strategies are evidence-informed rather than validated protocols.

## Prior presentation/redundant publication statement

Not applicable.

## Clinical trial registry

Not applicable – this is a narrative review of previously published literature and does not involve a clinical trial.

## Human and animal rights statement

Not applicable. This article is a narrative review of previously published literature and does not involve any studies with human participants or animals performed by any of the authors.

## Authorship criteria/contributor statement

All authors meet the journal's authorship criteria and have made substantial contributions to the conception and design of the review, literature evaluation and evidence synthesis, interpretation of the available evidence, manuscript preparation, critical revision, and final approval of the manuscript. All authors agree to be accountable for the integrity and accuracy of the work.

**Dr. Preeti Kulkarni**: Conceptualization of the review; development of the review objectives and overall framework; literature search and identification of relevant studies; evaluation and synthesis of the available clinical and biomechanical evidence; development of the clinical principles and chairside decision framework; preparation of tables and figures; manuscript drafting and preparation of the initial manuscript.

**Prof. Puneet Batra**: Methodological and scientific supervision of the review; refinement of the review framework; critical interpretation of the biomechanical and clinical evidence; evaluation of the clinical and orthodontic relevance of the findings; critical revision and substantive editing of the manuscript.

**Prof. Nikhillesh Vaiid**: Oversight of the overall scientific direction of the review; critical appraisal and interpretation of the evidence synthesis; evaluation of the clinical implications and evidence-to-action framework; critical review of the manuscript for scientific rigor, coherence, and contemporary relevance; substantive revision and preparation of the manuscript for submission.

## Authors' declaration

We confirm that this manuscript has been read and approved by all named authors, that the requirements for authorship have been met, and that each author believes the manuscript represents honest work.

## Human subjects statement

Not applicable. This article is a narrative review of previously published literature and did not involve human participants or identifiable human data.

## Animal subjects statement

Not applicable. This article is a narrative review of previously published literature and did not involve any studies on animals performed by the authors.

## Funding

This research did not receive any specific grant from funding agencies in the public, commercial, or not-for-profit sectors.

## Declaration of competing interest

The authors declare that they have no known competing financial interests or personal relationships that could have appeared to influence the work reported in this paper.
