# Supplementary material for: Improving the predictability of rotational correction with clear aligners: Evidence gaps, biomechanical principles, and clinical strategies
Source: J Oral Biol Craniofac Res. 2026 Oct 30;16(6):101567. doi: 10.1016/j.jobcr.2026.101567 (PMC13744331; doi:10.1016/j.jobcr.2026.101567)
Supplement: Supplementary file 1 [file mmc1.docx]

**Supplementary Table S1. Literature search and selection approach**

*Descriptive account of the narrative-review methodology; not a systematic-search protocol.*

| **Component** | **Approach used** |
| --- | --- |
| Review type | Narrative literature review with thematic synthesis; no registered protocol or formal systematic-review workflow. |
| Information sources | MEDLINE and Scopus; reference lists of relevant publications; additional relevant articles suggested by co-authors. |
| Search concepts | Clear aligner therapy; tooth rotation/derotation; attachments; staging; interproximal reduction; orthodontic biomechanics. |
| Search process | Exploratory searches using combinations of topic-related terms. No predefined, standardised Boolean strategy was applied across databases. Exact historical queries and retrieval counts were not retained. |
| Final search date | The exact date of the final database search was not retained. |
| Search limits and publication selection | No predefined publication-year or language filters were applied during database searching. During selection, emphasis was placed on recent, relevant evidence; the articles ultimately included were published from 2016 onwards. |
| Literature considered | Published, peer-reviewed journal articles and reviews relevant to rotational predictability, clinical management or underlying biomechanics. |
| Literature not systematically sought | Grey literature, including unpublished dissertations, conference abstracts and preprints; no separate grey-literature search was performed. |
| Selection and responsibility | The lead author evaluated relevance and decided which publications to include; co-authors suggested additional articles. No independent multi-reviewer screening or formal disagreement-resolution procedure. |
| Appraisal and synthesis | Qualitative critical reading of study design, findings and limitations; no standardised study-level risk-of-bias instrument. Evidence organised into clinical and biomechanical themes. |
| Evidence interpretation | Clinical studies informed achieved movement and treatment outcomes; laboratory and finite element analysis studies informed mechanistic interpretation, not direct clinical effectiveness. |
| Methodological limitations | Exploratory searching without preserved exact queries, retrieval counts, or a verified final search date limits reproducibility. Emphasis on recent literature may omit relevant earlier studies; omission of grey literature may miss unpublished or emerging findings. |
